# Modeling of cadmium(II) removal in a fixed bed column utilizing hydrochar-derived activated carbon obtained from discarded mango peels

**DOI:** 10.1038/s41598-022-11574-1

**Published:** 2022-05-14

**Authors:** Imran Khan Rind, Najma Memon, Muhammad Yar Khuhawar, Waheed Ali Soomro, Muhammad Farooque Lanjwani

**Affiliations:** 1 National Centre of Excellence in Analytical Chemistry, University of Sindh, Jamshoro, Pakistan; 2 Institute of Advanced Research Studies in Chemical Sciences, University of Sindh, Jamshoro, Pakistan; 3 Dr. M.A. Kazi Institute of Chemistry, University of Sindh, Jamshoro, Pakistan

**Keywords:** Pollution remediation, Sustainability

## Abstract

Cadmium is found in many underdeveloped countries' aquatic bodies. Therefore, contaminated water should be treated before consumption; henceforth, efficient and customized point-of-use filtration is foreseeable. Traditionally, carbon-based sorbents have been utilized for such treatments, but alternative sources are also being investigated. Hydrochars made from mango peels using a thermal activation process were employed as an adsorbent instead of activated carbon in this investigation. The prepared material was porous with active surface functionalities, and the interaction of cadmium with the surface was possibly ion-exchange in nature. The performance of a material for a candle water filtering system with a 2.5 cm internal diameter and a 30.48 cm column height was determined using the parameters acquired by the Thomas model. The material was found to be highly efficient at 453.5 L/min/Filter water, whereas 31670.6 L/min/Filter can be treated if the break point and exhaustion point are considered, respectively, as the candle replacement time. These findings indicate that activated hydrochar might be a suitable sorbent for removing cadmium ions from contaminated water.

## Intoduction

Water is life and necessary for social and economic development. It fulfills to maintain the integrity of the environment. The problems related to the quality and availability of water are pressing, where users of water resources are facing the main problems of water pollution that threaten the preservation of natural ecosystems. Industries generate contaminated wastewater that somehow reaches drinking water^1^. Metals widely contaminate the environment due to their inherent nonbiodegradable nature. Cadmium is a toxic metal and major pollutant found naturally in drinking water, foodstuffs, ores, and soil. WHO allows 0.003 mg/L as the limit of cadmium concentration in drinking water. Cadmium induces toxicity in human health, plants and animals and accumulates primarily in kidneys^2^.

Physical and chemical methods are employed to treat heavy metal contamination for drinking purposes. The most common traditional methods are membrane filtration, ion exchange, electrochemical treatment, chemical precipitation, solvent extraction and adsorption^3^. Adsorption is an important economical and practical alternate for water treatment that is viable and most commonly used in filtration systems, mainly in developing countries. Different adsorbents have been reported in the literature for metal removal contamination from drinking water, including resins^4^, zeolite^5^, biomaterials^6^, algal biomass^7^, kaolinite^8^, and peanut hulls^9^. However, activated carbon is the most versatile and can remove a wide variety of compounds to nearly undetectable levels and is therefore used in candle filters and other filtration units. Activated carbon has a microcrystalline carbon nature with very high surface area and porosity^10^ and is produced by pyrolyzing and activating hardwood (lignin type) or wastes such as coconut shell, bonechar, etc. Soft biomass (lignocelluosic type) is abundant on earth and may be converted to activated carbon, but the process of direct pyrolysis results in a very low yield.

The production of solid carbonaceous materials known as hydrochar from wet biomass can be carried out by a hydrothermal carbonization (HTC) process. Hydrochar formation occurs in the presence of water as a reaction medium at temperatures ranging from 180 to 250 °C and autogenous pressure to give products, namely, solid carbonaceous, water soluble organic fractions (organic acids and sugar), with the release of CO_2_ gas^11^. Hydrochar has chemical characteristics and a physical structure comparable to lignite coal^12^. The presence of a high surface area and diverse surface sites with highly oxygenated functional groups, such as carbonyl, carboxylic and hydroxyl groups, on hydrochar make it a suitable precursor for the production of highly porous activated carbon^13^. Activated carbons are prepared from different biomasses, such as wood, agricultural waste, sunflower shells, wheat straw, cassava peel, pomegranate peel, orange peels and coffee waste. Biomass binds heavy metal ions with reusability of biomaterials and low operating cost. These are widely used to remove metal contamination from water^14^.

Fruit peel waste is generated in a large amount by juice shops and industries. The material contains a high amount of pectin in which carboxylate groups receive more scientific attention. The mango family (*Mangifera indica* L) is one of the delightful fruits grown in tropical and subtropical areas. According to the FAO, approximately 50.6 million tons of mango are produced annually, and a large amount of waste is produced upon consumption of the fruit^15^. Biomass (mango peels) contains different organic compounds, such as lignin, cellulose and hemicelluloses, and is rich in hydroxyl and carboxylic groups that can bind heavy metal ions^16^. Many researchers have worked on continuous fixed-bed column experiments using different adsorbents for the removal of cadmium ions from water ^17–20^. Fixed bed studies are suitable for scaling up the sorptive removal process for possible point-of-use filtration columns.

In a reported work, activated hydrochar prepared from mango peels was tested to remove lead ions from contaminated water. The equilibrium uptake capacity was 38.31 mg/g with 88.70% removal of lead ions from the fixed-bed column. Activated hydrochar is a suitable sorbent to remove lead ions from drinking water samples^21^.

In this work, peels of mango were used for the preparation of activated hydrochar, and a continuous fixed-bed column method was used for the removal of cadmium ions from polluted water. The effects of different column parameters, such as bed heights, linear flow rates and cadmium concentrations, were investigated during the adsorption process. An equilibrium adsorption study was carried out using Langmuir and Freundlich linear isotherm models, and a kinetic study of cadmium adsorption was also performed. Breakthrough curve adsorption experimental data of cadmium were modeled with fixed-bed column models Thomas, Yon-Nelson and Adam-Bohart. Furthermore, to understand the practical viability of this material, parameters obtained by the best fitted model were extrapolated to calculate the volume of water that could be treated with one candle filter having activated hydrochar as the sorbent.

## Results and discussion

### Characterization of activated hydrochar

Fifteen grams of wet mango peels produced only a 2 g (13.3%) yield of hydrochar. Hydrochar was thermally treated at 400 °C, 600 °C and 800 °C to produce activated hydrochar, but the prepared activated hydrochar at 400 °C indicated better removal efficiency for cadmium therefore its results have been included. The yield was obtained 44.63% (Table 1). During activation of hydrochar, the % yield decreased because colloidal and low-density carbon evaporated into soot ^22^. The physicochemical characteristics of hydrochar and activated hydrochar were tested for moisture content, ash content, total acid density, methylene blue number and iodine number. The results of the physico-chemical characteristics are mentioned in Table 1. The activated hydrochar with a lower moisture content than hydrochar showed a better quality for the adsorption process. Ash is said to be the residue when material is burned off and was found to be 3.8 and 2.16% for hydrochar and activated hydrochar, respectively. The methylene blue numbers were 909 and 909.09 mg/g for hydrochar and activated hydrochar, respectively, indicating that the samples were mesoporous^23^. The iodine numbers for hydrochar and activated hydrochar were 751.73 and 869.01 mg/g, respectively. The results showed a high adsorption capacity for activated hydrochar and micropore networks^24^. The measurement of the total acid density before and after activation of activated hydrochar was found to be 0.15 and 0.1 mmol/g. Acid density decreased with increasing temperature because during the formation of hydrochar and activated hydrochar, carbon dioxide was eliminated, and aromatic character increased, which led to the decomposition of acidic oxygenated functional groups.

**Table 1:** Physico-chemical characteristics of the hydrochar and activated hydrochar.

Parameters	Hydrochar (HTC)	Activated hydrochar (ACH)
Yield (%)	13.3	44.63
Moisture (%)	13.29	5.08
Ash (%)	3.8	2.16
Methylene blue number (mg/g)	909	909.09
Iodine number (mg/g)	751.73	869.01
Acid density (mmol g^−1^)	0.15	0.1
Surface area (m^2^/g)	8.266	3.711
Pore volume (c.c./g)	0.007	0.009
Pore diameter (nm)	3.065	6.282

**Figure 1:**
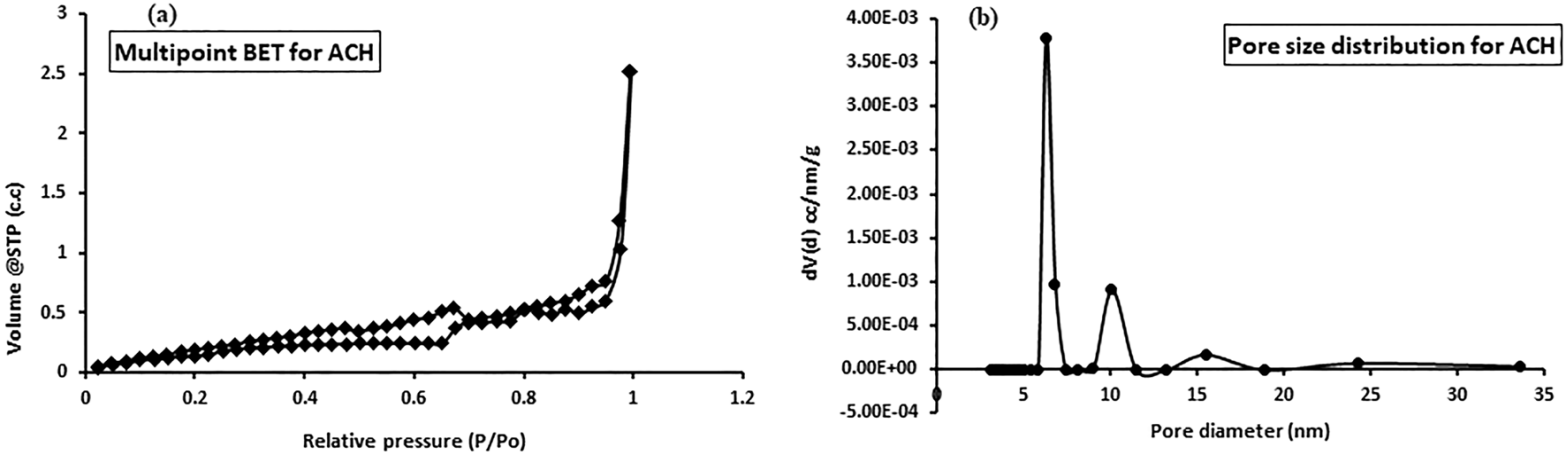
Nitrogen adsorption–desorption isotherm (**a**) and pore size distribution of activated hydrochar (**b**).

The surface areas of hydrochar and activated hydrochar were found to be 8.266 and 3.711 m^2^/g, respectively, while pore volumes were 0.007 and 0.009 c.c./g, respectively (Table 1). Low surface area and pore volume were found due to thermal activation. The pore diameters calculated by the BJH method were 3.065 and 6.282 nm (Table 1). Figure 1a shows the IUPAC classification type III H3 hysteresis loop in the adsorption–desorption isotherm with unrestricted multilayer formation and dense particles with slit-like pores^25,26^. The size less than 20 nm for activated hydrochar conformed to the mesoporous structure, as indicated in Fig. 1b.

**Figure 2:**
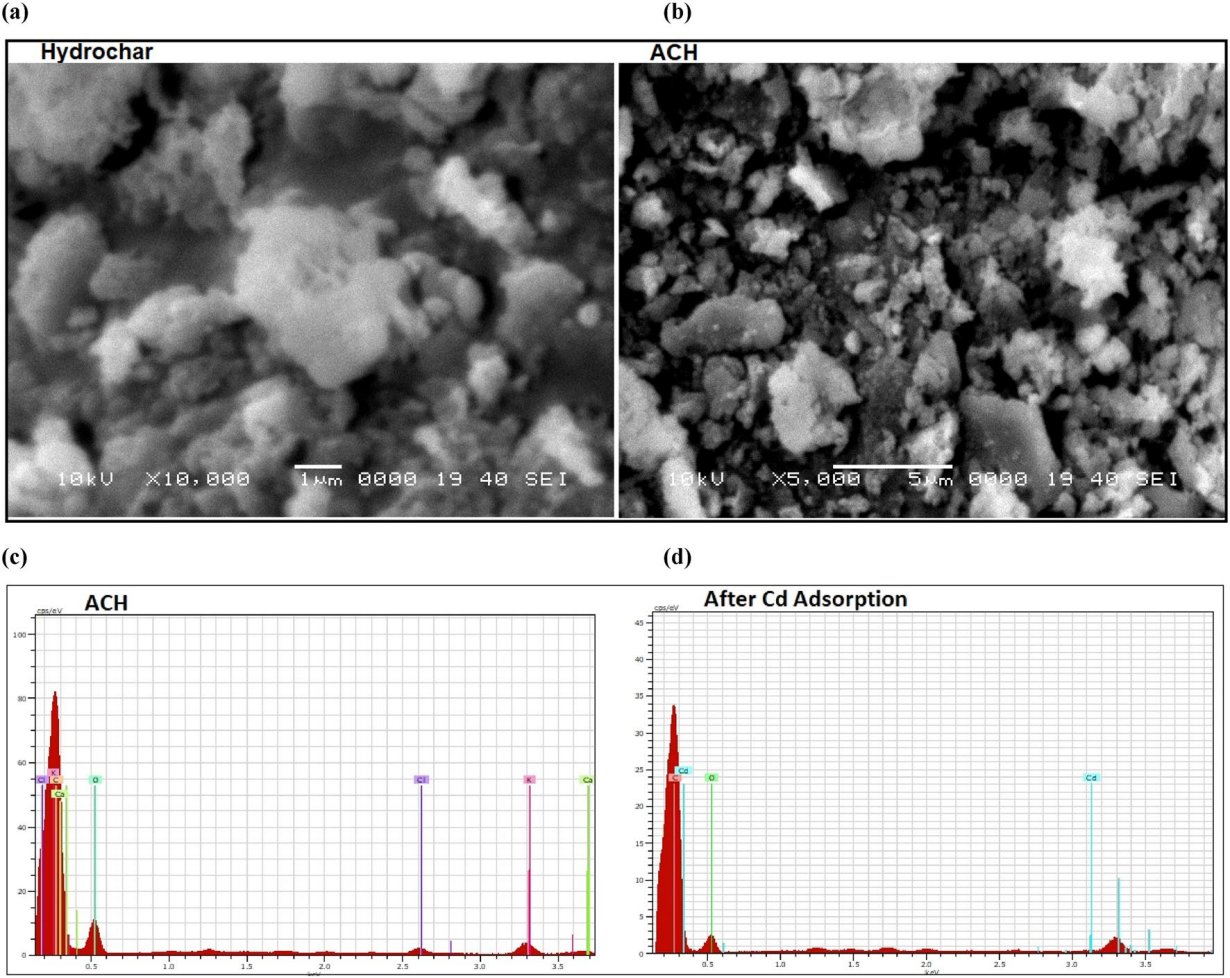
SEM images of hydrochar (**a**), ACH (**b**) and EDS spectra before (**c**) and after Cd adsorption (**d**).

The surface morphology before and after activation is explained by SEM images, as shown in Fig. 2a-b. A wide range of shapes and particle sizes were found for hydrochar and activated hydrochar. Surface morphology changed after activation of hydrochar. As shown in Fig. 2, hydrochar particles are globular agglomerated, while after activation, hydrochar particles are interconnected and condensed (Fig. 2). Energy dispersive X-ray spectroscopic measurements show a high percentage of carbon after thermal activation of hydrochar (Fig. 2c), and cadmium is adsorbed with activated hydrochar, as indicated in Fig. 2d.

**Figure 3:**
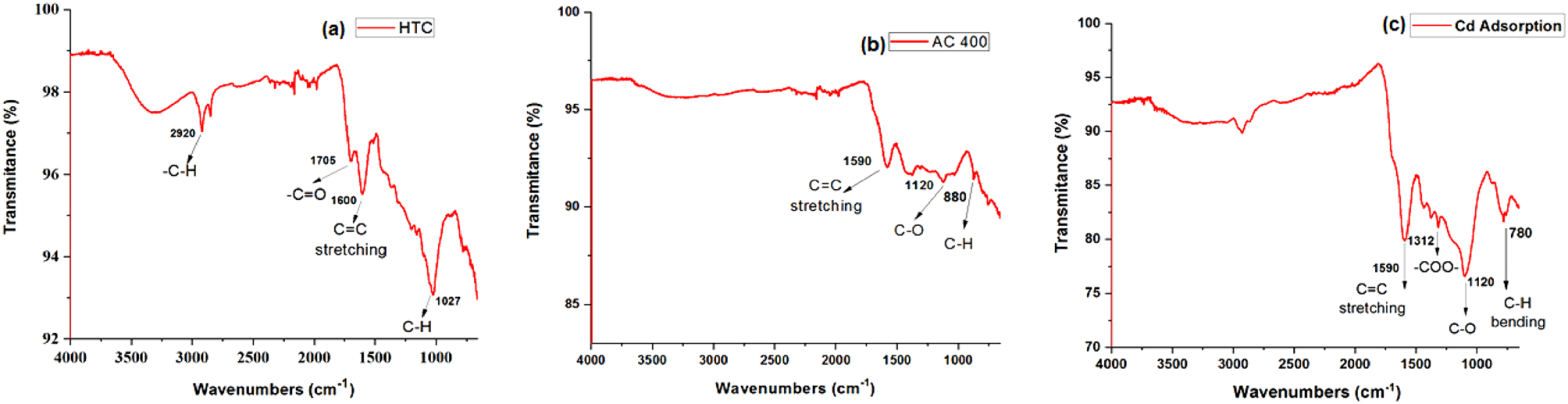
FTIR frequencies of hydrochar (**a**), activated hydrochar (**b**) and Cd-adsorbed activated hydrochar (**c**).

FTIR spectra were used to identify functional groups present on the surface before and after activation of activated hydrochar. Figure 3a-c represents the possible band frequencies in the FTIR spectrum present on the surface of hydrochar, activated hydrochar and cadmium adsorb activated hydrochar. The hydrochar peak at 2920 cm^–1^ (Fig. 3a) was attributed to the C–H stretching vibrations of –CH_3_ and –CH_2_ groups in the hydrochar structure. This band completely disappears after activation of hydrochar (ACH), showing decomposition of hemicellulose. Two peaks assigned at 1700 cm^–1^ and 1600 cm^–1^ were attributed to the carbonyl stretching vibration of –COO and C=C stretching, and the latter was attributed to the aromatic ring in the lignin. A peak observed in activated hydrochar (Fig. 3b) at 1590 cm^–1^ was allotted to the (C=C) stretching vibration. Two peaks appeared at 1120 cm^–1^ and 880 cm^–1^, expressive of C–O in hydroxyl groups and C–H vibrations in planes and out of plane. A peak observed at 1312 cm^–1^ in activated hydrochar after cadmium adsorption (Fig. 3c) was allotted to the symmetric stretching vibration of –COO. The observed spectrum supported the presence of oxygenated functional groups that were responsible for cadmium adsorption.

### Adsorption isotherms and kinetic studies

**Figure 4:**
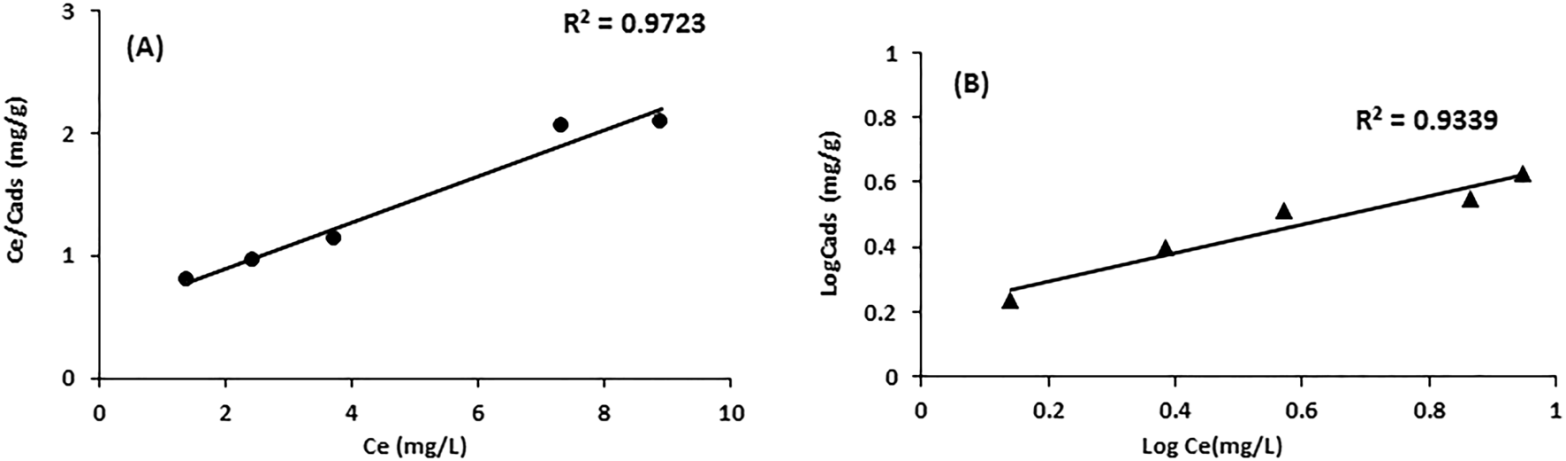
Langmuir (**a**) and Freundlich **(b)** isotherm models of cadmium ions.

The linear form of the Langmuir isotherm was plotted between Ce/Cads (mg/g) vs Ce (mg/L) to obtain a straight line, as shown in Fig. 4a. Parameter values were evaluated using the slope and intercept of the plot, as given in Table 2. The linear form of the Freundlich isotherm model was plotted between log Ce (mg/L) and log Cads (mg/g) (Fig. 4b). The correlation coefficient for the Langmuir isotherm (0.9723) was significantly higher than that for the Freundlich isotherm for Cd^2+^ (0.9339), which indicated that the adsorption data were well described by the Langmuir isotherm. A good fit to the experimental data of the equation reflects that the adsorbate has monolayer coverage of sites and is homogeneous on the adsorbent surface.

**Table 2:** Isotherm parameters for the sorption of cadmium ions on activated hydrochar.

Isotherm model	Parameters	Cd^2+^
Langmuir isotherm	Q_o_ (mg/g)	52.87
	b (L/mg)	0.0097
	R^2^	0.9723
	R_L_	0.91
Freundlich isotherm	K_f_ (mg/g)	1.63
	n	2.29
	1/n	0.43
	R^2^	0.9339
Dubinin-Radushkevich (D–R)	Xm (mg/g)	22.93
	E (kJ/mol)	1.84
	R^2^	0.9478

**Figure 5:**
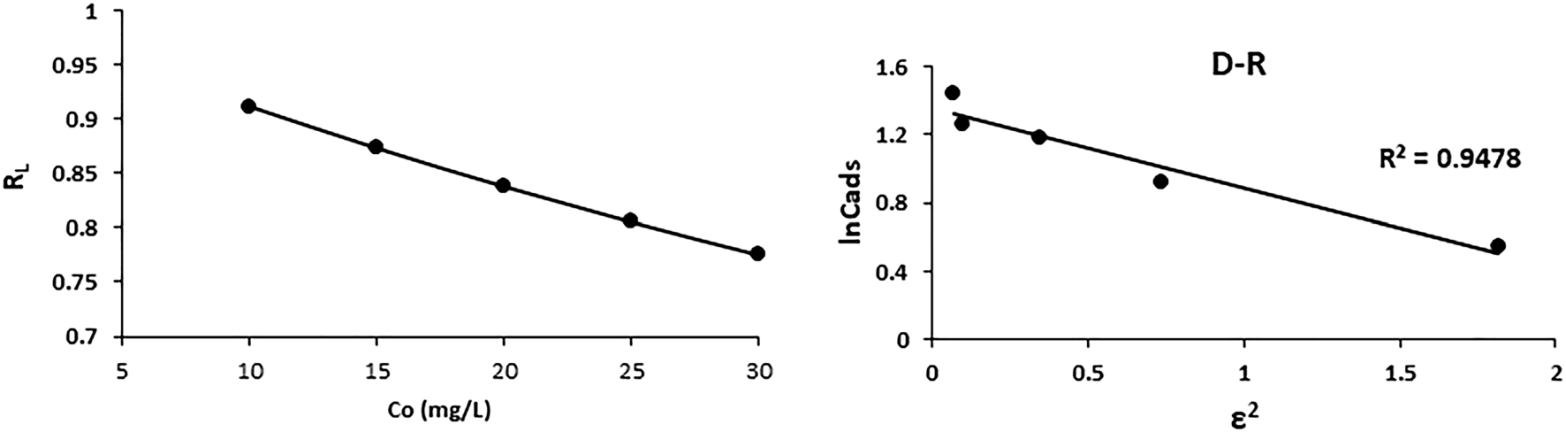
Separation factor against initial cadmium concentration and D–R isotherm model of cadmium ions.

For the Langmuir adsorption study, the isotherm shape was categorized by the dimensionless constant separation factor (R_L_). Figure 5 shows the calculated R_L_ values at various initial cadmium concentrations. R_L_ values ranging from 0–1 confirm the favorable uptake of cadmium adsorption, and the R_L_ range at higher cadmium concentrations shows that cadmium adsorption is more favorable. The degree of favorability is related to the irreversibility of the system and describes the qualitative assessment of adsorbent-activated hydrochar and cadmium interactions.

The Dubinin –Radushkevich (D –R) isotherm model differentiates the chemical and physical adsorption mechanisms. A linear graph of the D–R isotherm was obtained with a correlation coefficient of R^2^ = 0.9478 (Fig. 5) when plotted between $\varepsilon^{2}$ and lnCads (mg/g). Table 3 also shows the results of the D–R adsorption model, where the Xm and E values were found to be 36.74 (mg/g) and 1.84 (kJ/mol), respectively, showing that the adsorption process is considered physical in nature.

**Table 3:** Kinetic parameters for the adsorption of cadmium on activated hydrochar.

Isotherm model	Parameters	Cd^2+^
Pseudo first-order	qe (mg/g)	0.95
	k_1_ (minˉ^1^)	0.06
	R^2^	0.9796
Pseudo second-order	qe (mg/g)	49.02
	k_2_ (mg minˉ^1^)	0.0067
	R^2^	0.9997

**Figure 6:**
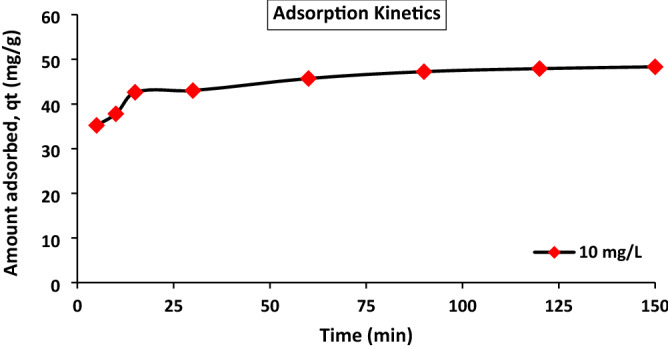
Kinetic equilibrium adsorption of cadmium ions on ACH (dose 0.03 g, cadmium conc. of 10 mg/L).

Kinetic study is an important physicochemical assessment for the basic traits of adsorbent quality. The cadmium amount adsorbed at various intervals of time using a cadmium concentration of 10 mg/L is indicated in Fig. 6. The time required to reach equilibration was approximately 120 min. The adsorption capacity of cadmium ions increased with increasing time and attained maximum equilibrium capacity at 120 min, indicating that no more cadmium was removed from the solution.

**Figure 7:**
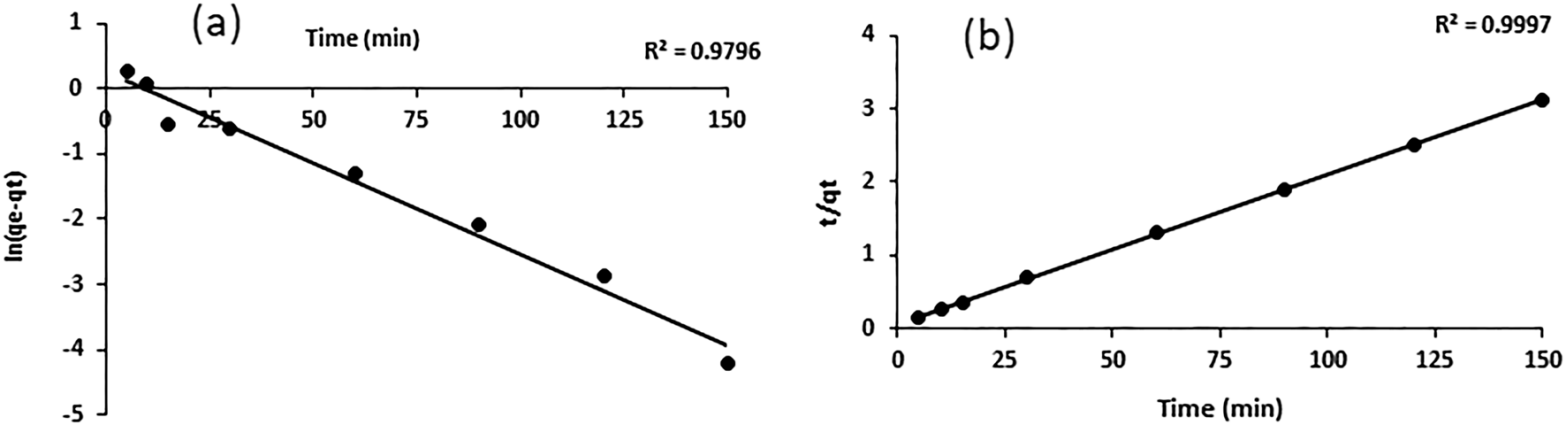
Kinetic adsorption data of pseudofirst-order (**a**) and pseudosecond-order (**b**).

Pseudofirst- and pseudosecond-order kinetic parameter values are given in Table 3. The equilibrium uptake value and higher correlation coefficient (0.9997) proved that the adsorption data were well fitted with pseudosecond-order kinetics for the whole adsorption time, as mentioned in Fig. 7a and 7b.

## Adsorption of cadmium ions in fixed-bed column

### Effect of bed heights

**Figure 8:**
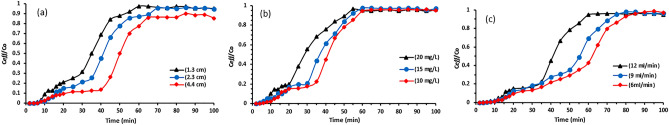
Effects of bed heights (**a**), cadmium concentrations (**b**) and flow rates (**c**) on breakthrough curves of cadmium adsorption on ACH.

Different bed heights of 1.3, 2.4 and 4.4 cm were utilized to determine the breakthrough curve of cadmium ions using a flow rate of 12 mL/min and cadmium concentration of 10 mg/L. When the bed height increased from 1.3 to 4.4 cm, the breakthrough time and saturation time also increased (Fig. 8a). A longer bed height reduced the effluent concentration and delayed the saturation time of the adsorbent (Table 4). A longer bed was able to operate for a longer time, and a smaller bed saturated earlier due to more binding sites and a large surface area available for metal adsorption^27^.

**Table 4:** Description of column parameters of cadmium ions on activated hydrochar.

Parameters	Z (cm)	Co (mg/L)	Q (mL/min)	t_B_ (min)	q_*eq*_ (mg/g)	Total removal (%)
Effect of bed height	**1.3**	10	12	12	4.19	87.50
	**2.4**	10	12	16	3.39	88.38
	**4.4**	10	12	25	2.66	88.66
Effect of concentration	2.4	**10**	12	16	3.39	88.38
	2.4	**15**	12	14	4.49	89.28
	2.4	**20**	12	10	4.82	90.00
Effect of flow rate	2.4	10	**6**	25	2.63	87.33
	2.4	10	**9**	20	3.14	87.22
	2.4	10	**12**	16	3.39	88.38

Significant values are in bold.

### Effect of concentrations

The effects of different cadmium concentrations (10, 15 and 20 mg/L) on the breakthrough curve were examined by using a 12 mL/min flow rate and 2.4 cm bed height. The breakthrough curve was greatly affected when the concentration increased from 10 to 20 mg/L (Fig. 8b). With increasing cadmium concentration, the break time and saturation time decreased. At lower concentrations, the breakthrough time increased mainly due to lower mass transfer during the adsorption process, and more cadmium volume was needed for treatment. The saturation time decreased with increasing cadmium concentration due to the greater adsorbate volume per unit surface area of the activated hydrochar, which caused saturation earlier. The adsorption capacity increased with increasing cadmium concentration (Table 4) due to faster saturation of the activated hydrochar^28^.

### Effect of flow rates

Continuous fixed-bed column experiment flow rates influenced the removal capacity of cadmium. The effects of different flow rates of 6, 9 and 12 mL/min were studied for the removal of cadmium ions with bed heights and metal concentrations of 2.4 cm and 10 mg/L, respectively. The breakthrough time and exhaustion time increased with decreasing flow rate from 12 mL/min to 6 mL/min (Fig. 8c). The contact time of cadmium solution decreases with a high flow rate and has less contact with the adsorbent to diffuse. The residence time of the solution was larger at a lower flow rate and allowed to diffuse into pores. The adsorption capacity increased from 2.63 to 3.39 mg/g as the flow rate increased (Table 4).

## Adsorption modeling

### Adam–Bohart model

**Figure 9:**
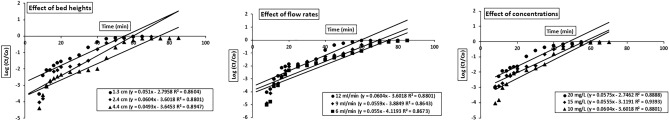
Adam–Bohart model linear fitting curve for bed heights, flow rates and concentrations. The dots indicate experimental results while the lines show model fit.

The initial part of the breakthrough curve during the adsorption process is predicted in this model. Parameters such as the Adam–Bohart rate constant (*k*_*AB*_) and maximum saturation concentration of the metal (*No*) were studied with bed depth, concentration, and flow rate. *k*_*AB*_ and *No* values were obtained by linear plotting of log (Ct/Co) vs time (Fig. 9). The values did not decrease with increasing bed height due to the availability of more binding sites for metal adsorption (Table 5). When the concentration was increased, the activated hydrochar in the column was exhausted as the concentration loading was higher. This condition was also the same for increasing flow rates. The solution volume entering the column was higher with a high flow rate and caused the adsorbent to saturate earlier^27^.

**Table 5:** Adam–Bohart model parameters for the adsorption of cadmium ions.

Bed height Z (cm)	Co (mg/L)	Flow rate (mL/min)	K_AB_ × 10^–3^ (L/min mg)	*N*_o_ (mg/L)	R^2^
**1.3**	10	12	5.10	6449.37	0.8604
**2.4**	10	12	6.04	3727.03	0.8801
**4.4**	10	12	4.90	2565.31	0.8947
2.4	**10**	12	6.04	3727.03	0.8801
2.4	**15**	12	3.70	5268.75	0.9393
2.4	**20**	12	2.88	5970.00	0.8880
2.4	10	**6**	5.50	2416.01	0.8673
2.4	10	**9**	5.59	3309.39	0.8643
2.4	10	**12**	6.04	3727.03	0.8801

Significant values are in bold.

### Thomas model

**Figure 10:**
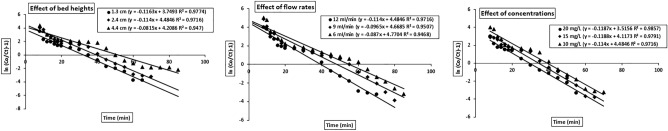
Thomas model linear fitting curve for bed height, flow rates and concentrations. The dots indicate experimental results while the lines show model fit.

This model predicted breakthrough curve results. Thomas parameters with maximum solid phase concentration in mg/g and kinetic coefficient in k_TH_ were obtained by plotting data in linearized form that described the column behavior. The k_TH_ and *qo* values were determined by a linear plot of ln[(Co/Ct)-1] vs time (Fig. 10). The *qo* values decreased with increasing adsorbent amount and increased with increasing flow rate and concentration (Table 6) because the driving force amount was high enough for metal adsorption. A larger amount of cadmium was perforated in activated hydrochar^29^.

**Table 6:** Thomas model parameters for the adsorption of cadmium ions.

Bed height Z (cm)	Co (mg/L)	F. rate (mL/min)	K_TH_ × 10^–3^ (mL/min mg)	*q*_o_ (mg/L)	R^2^
**1.3**	10	12	11.63	12.89	0.9774
**2.4**	10	12	11.4	9.44	0.9716
**4.4**	10	12	8.15	6.19	0.947
2.4	**10**	12	11.4	9.44	0.9716
2.4	**15**	12	7.92	12.48	0.9791
2.4	**20**	12	5.94	14.22	0.9857
2.4	10	**6**	8.70	6.58	0.9468
2.4	10	**9**	9.65	8.71	0.9507
2.4	10	**12**	11.4	9.44	0.9716

Significant values are in bold.

### Yoon–Nelson model

**Figure 11:**
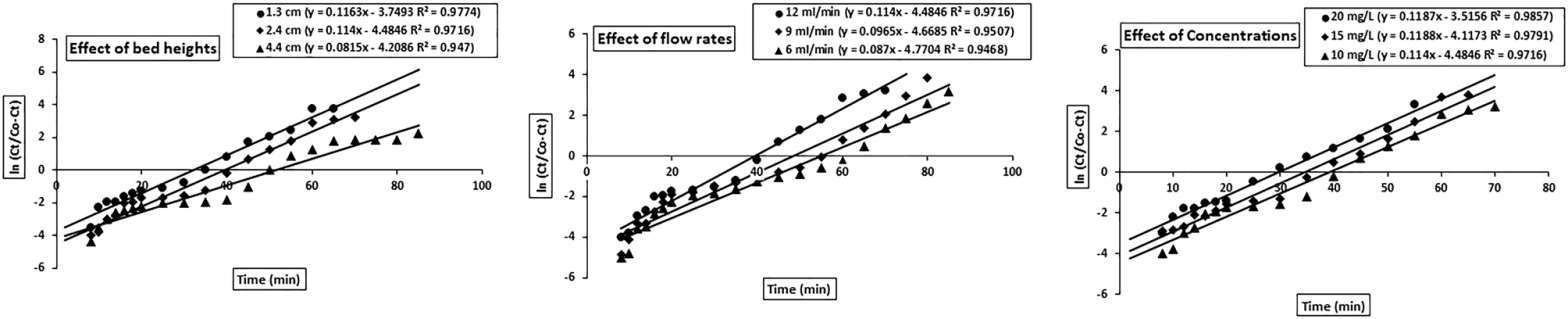
Yoon–Nelson model linear fitting curve for bed heights, flow rates and concentrations. The dots indicate experimental results while the lines show model fit.

Breakthrough curve activities were predicted by applying the Yoon-Nelson model. It is applicable to a single component system, and fewer data are needed to construct model values. The linearized form of this model predicted a 50% adsorbate breakthrough time ($\tau )$ and rate constant (k_YN_). The $\tau$ and k_YN_ values were obtained by a linear plot of ln[(C_t_/(Co–C_t_)] vs time (Fig. 11). Higher values of k_YN_ were obtained with increasing metal concentration and flow rate and smaller with increasing bed height (Table 7) because it increased the force and controlled the mass transfer reaction in solution. The 50% breakthrough time ($\tau )$ decreased with increasing concentration and flow rate, indicating that the column exhausted rapidly^30^.

**Table 7:** Yoon–Nelson model parameters for the adsorption of cadmium ions.

Bed height Z (cm)	Co (mg/L)	F. rate (mL/min)	K_YN_ × 10^–3^ (min^–^)	$\tau$(min)	R^2^
**1.3**	10	12	9.69	32.24	0.9774
**2.4**	10	12	7.13	39.34	0.9716
**4.4**	10	12	3.26	51.64	0.947
2.4	**10**	12	7.13	39.34	0.9716
2.4	**15**	12	8.48	34.66	0.9791
2.4	**20**	12	11.87	29.62	0.9857
2.4	10	**6**	3.48	54.83	0.9468
2.4	10	**9**	4.83	48.38	0.9507
2.4	10	**12**	7.13	39.34	0.9716

Significant values are in bold.

Based on the column model results, Thomas and Yoon-Nelson provided a good correlation range (R^2^ 0.947–0.9857) for the prediction of the breakthrough curve study and showed good agreement with the experimental data.

In the adsorption process, Thomas model was the best fitted model where internal and external mass diffusion are not the limiting step and is described by a pseudo second-order reaction rate which reduces a Langmuir isotherm at equilibrium. The axial and radial dispersion in the fixed bed column is negligible. The Thomas model optimization study was also applied to a candle water filtration system that contained a 2.5 cm internal diameter and 30.48 cm height of the column. Approximately 453.4375 L/min/Filter amount is yielded in a break point and 31,670.625 L/min/Filter during column exhaustion time. Therefore, water treatment facilities can easily access water free of cadmium contamination when using activated hydrochar in place of commercially available activated carbon in candle filters.

The Yoon-Nelson model predicted a 50% column breakthrough time and enabled exhaustion time without any time spent for the experimental study. The Adam–Bohart model predicted the maximum saturation concentration and supplied data to apply experiments for large-scale effluent water treatment systems, but this required additional calculations and parameters to obtain modeling data. Adam-Bohart followed reversible pseudo second-order kinetics, and no axial dispersion occurred during the adsorption process.

## Effect of matrix ions

The interference of calcium, magnesium, sodium and potassium to remove cadmium ions was assessed under optimized conditions in a fixed-bed column experiment. Sodium, potassium, calcium and magnesium ions of 100 and 500 mg/L solution were added individually to a 10 mg/L cadmium ion solution. Cadmium ions were still removed by more than 90% with a relative error not higher than 5%, and there was no interference on the removal of cadmium ions even in the presence of high concentrations of alkali and alkaline earth metals (Table 8).

**Table 8:** Effect of matrix ions on the removal of cadmium ions in the column.

Metal	Added as	Conc. (mg/L)	Conc. After adsorption	Removal (%) (RSD %)
Cadmium	–	10	0.40	96 (0.3)
Sodium	NaCl	100	4.534	95.47 (1.1)
		500	3.055	99.39 (0.6)
Potassium	KCl	100	4.707	95.29 (1.7)
		500	4.29	99.14 (0.8)
Calcium	CaCl_2_	100	6.532	93.47 (2.1)
		500	8.33	98.33 (0.4)
Magnesium	MgCl_2_	100	4.434	95.57 (1.6)
		500	6.372	98.73 (1.3)

## Real water application

### Removal of spikes from real water matrix

Cadmium ions were removed after spiking 10 mg/L cadmium into the groundwater, tap water and river water samples. The samples were passed under optimized conditions through a fixed column with a 2.4 cm bed height and 12 mL/min flow rate. The effluent concentration was analyzed by flame atomic absorption spectrometry. The results are mentioned in Table 9. Cadmium 69.37–94.92% was removed with an RSD of 0.4–1.2% from the samples after spiking with 10 mg/L.

**Table 9:** Cadmium ion removal from a real water matrix after spiking cadmium.

Matrix	Spiked amount of Cd^2+^ (mg/L)	Residual Conc. of Cd^2+^ (mg/L)	Removal (%) (RSD n = 3)
Groundwater	10	0.508	94.92 (0.6)
Tap water	10	1.321	86.79 (1.2)
River water	10	3.063	69.37 (0.4)

### Removal of cadmium from groundwater samples

Groundwater samples were collected from district Matiari, Sindh, Pakistan from hand pumps and motor pumps and were examined for physicochemical parameters pH, electrical conductivity (EC) and total dissolved solids (TDS) for possible tolerance effect as results are given in Table 10. The pH value determined the strength of acidity or alkalinity of the water solution, while EC and TDS indicate the ionic concentrations, due to geological weathering conditions with acquiring concentrations of the dissolved minerals in the water. The collected samples were passed into the fixed-bed column using optimized conditions. Samples were contaminated with cadmium with a range higher than the WHO limit of 0.003 mg/L. After passing into the column, activated hydrochar removed cadmium ions 91.03–96.36% with an RSD of 0.2–1.4% from the water samples to bring the cadmium concentrations within the WHO limit.

**Table 10:** Cadmium ions removal from groundwater samples.

S #	Sample location	pH	EC µS/cm	TDS mg/L	Initial Conc. of Cd^2+^ (mg/L)	Residual Conc. of Cd^2+^ (mg/L)	Removal (%) (RSD n = 3)
1	Saeedabad city	7.23	1450	928	0.004	0.0003	92.5 (1.4)
2	Village Seerachu Kaka	7.48	1227	785	0.0055	0.0002	96.36 (0.5)
3	Village Sher Muhammad Thora	6.67	1591	1018	0.0078	0.0007	91.03 (0.2)

## Desorption study

**Figure 12:**
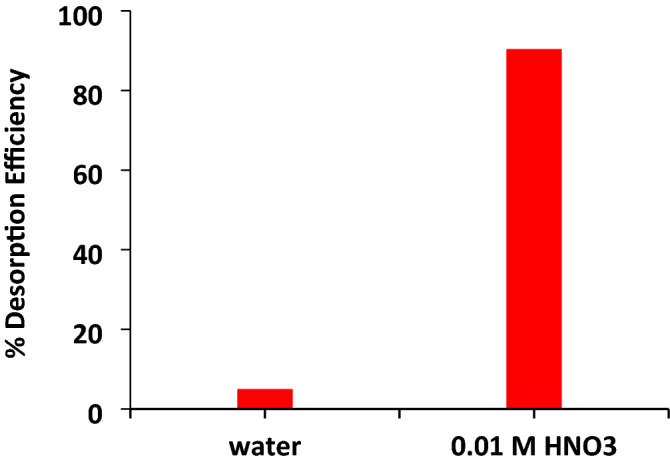
Desorption experiment for cadmium ions.

Desorption of cadmium from activated hydrochar using deionized water was very negligible (5%) compared to 0.01 M HN0_3,_ which was more effective with a 90.4% desorption efficiency (Fig. 12). Activated hydrochar is stable in the environment due to its chemical stability and physicochemical properties. The exhausted activated hydrochar was regenerated by putting HNO_3_ at room temperature (30 ± 2 °C) on a thermal shaker for 4 h at 200 rpm. The regenerated activated hydrochar was examined for the removal efficiency of cadmium and was observed 92% as compared to fresh activated hydrochar 97.07%. The results indicated a reasonable removal efficiency of recovered activated hydrochar.

## Conclusions

In a fixed-bed column experiment, thermally activated hydrochar made from mango peels was successfully used to remove cadmium from aqueous solution. The equilibrium experimental results were consistent with the Langmuir isotherm and yielded an adsorption capacity of 52.87 mg/g, indicating that the adsorbate formed monolayer coverage and that the sites on the adsorbent surface were homogeneous, while the kinetic experimental results were consistent with pseudosecond-order kinetics. Experimental study revealed that cadmium removed 88.38% from aqueous solution with an adsorption capacity of 3.39 mg/g using a 2.4 cm bed height, 12 mL/min flow rate and 10 mg/L metal concentration. The experimental breakthrough curves results obtained by adsorption process follows Thomas model which tells that the sorption of cadmium is reversible pseudo second order in nature without any axial dispersion and is instantaneous. The Thomas model yielded a 9.44 mg/g adsorption capacity. The material was discovered to be extremely efficient, capable of treating 453.5 L/min/mg water if the break point is used as the replacement time, and 31,670.6 L/min/mg if the exhaustion point is used as the replacement time. Furthermore, the cadmium was removed even in real water and in the presence of other common cations in high concentration.The study revealed that activated hydrochar has the potential to remove cadmium ions from contaminated water.

## Materials and methods

### Biomass collection

Sindhri waste mango peels collected from juice shops of Hyderabad, Sindh, Pakistan were used for the preparation of activated hydrochar. Mango peels were chopped and frozen at − 20 °C in an oven until use. Powdered material was sieved, and a particle size of 0.8–1.0 mm was obtained.

### Groundwater samples collection

Groundwater samples were collected from Matiari district used for drinking purposes. The water samples were filtered and preserved with HNO_3_. Cadmium ions were analyzed in samples before and after adsorption by using a Flame Atomic Absorption Spectrometer (PerkinElmer AAnalyst800, Singapore) using a fixed-bed column experiment after passing a 20 mL water sample using an optimized bed height and flow rate of 2.4 cm and 12 mL/min.

### Preparation of standard cadmium (II) solution

Analytical reagent grade chemicals were used in the experiments. A cadmium stock solution of 1000 mg/L was prepared by dissolving a proper amount of cadmium nitrate in distilled water. Solution-required cadmium concentrations were obtained by diluting with distilled water.

### Synthesis of hydrochar from biomass

The biomass was prepared by a reported procedure^21^ as follows: 15 g of fresh biomass (mango peels; var. Sindhri) was transferred into quartz vials, placed into Teflon lined stainless steel autoclaves and heated for five hours at 220 °C in an oven to obtain carbon-enriched material known as hydrochar. The autoclave was cooled, and hydrochar material was recovered by filtration. Hydrochar material was washed with distilled water continuously until neutral and colorless filtrate was attained. The hydrochar material was finally dried overnight at 60 °C in an oven.

### Activation of hydrochar

A thermal activation process was carried out for the synthesis of activated hydrochar (sample coded as ACH) from hydrochar. One hundred grams of hydrochar was heated at 400 °C, 600 °C and 800 °C in a muffle furnace for 2 h under a 180 mL/min flow rate of N_2_ gas. Activated hydrochar was cooled in a furnace with a flowing N_2_ atmosphere to 60 mL/min. Activated hydrochar yield was calculated using Eq:

1 $${\text{ACH Yield }}\left( {{{wt\% }}} \right){ } = { }\frac{{\text{Weight of ACH}}}{{\text{Weight of hydrochar}}}{ } \times 100{ }$$

### Characterization

Characterization of the structure and composition of activated hydrochar before and after activation was carried out as follows. The total ash content before and after activation was determined by ASTM method D 2866 (2016). The moisture contents for hydrochar and activated hydrochar were calculated using ASTM method D 2867. The total acid density parameter for activated hydrochar was measured by the acid–base back titration method using the indicator phenolphthalein^31^.

Total acid density was calculated using equation:

2 $${\text{C}}\left( {\text{H}} \right) \, = {\text{ C}}\left( {{\text{OH}}} \right) \, *\Delta V/ m$$

where V is the titrant volume in mL, OH represents the molar concentration of sodium hydroxide and m represents the mass of the substance in grams.

Methylene blue number and iodine number were used to determine the adsorption capacity of hydrochar and activated hydrochar samples. The methylene blue number measures the amount of mesopores of the activated hydrochar. A Langmuir isotherm adsorption experiment was carried out to determine the methylene blue number^21,32^. The amount of methylene blue adsorbed at equilibrium (q_e_) in mg/g was measured by Eq:

3 $$qe = \frac{{\left( {Co - Ce} \right). V}}{M}$$

where Co is the initial concentration and Ce is the methylene blue equilibrium concentration in mg/L. V and M represent the treated volume in litter and mass of the substance in grams. The maximum amount adsorbed for each sample was evaluated by using the Langmuir model, calculated by the following equation:

4 $$\frac{Ce}{{qe}} = \frac{ 1}{{Qob}} + \frac{Ce}{{Qo}}$$

where Q_0_ and b represent the Langmuir monolayer adsorption capacity (mg/g) and equilibrium constant expressed (L/mg), respectively. Q_0_ and b values were calculated graphically by plotting Ce/qe vs Ce. It gives a straight line of slope 1/Q_0_, which relates to maximum monolayer capacity in mg/g and intercept of 1/Q_0_b.

The iodine number also measures the adsorption capacity and expresses the micropore content of the material. It is determined by ASTM method (2016) D4607. It was defined as the amount adsorbed in mg/g of carbon substance.

The surface morphology of hydrochar and activated hydrochar was observed by scanning electron microscopy (SEM) using model JSM-6491 LV, Joel. Japan. The materials were coated with Au to induce electroconductivity. Samples for SEM morphology were prepared by dispersing 20 mg of each sample in 20 mL of distilled water and then dried at 70 °C overnight in an oven. Samples were then characterized at the Metallurgy and Material Department laboratories, Mehran University of Engineering and Technology Jamshoro, Sindh, Pakistan.

Energy dispersive X-ray spectroscopy (EDX) (Oxford Penta Fetx 5) equipped with SEM was used to measure elemental analysis before and after adsorption of cadmium.

Fourier transform infrared spectroscopy (FTIR) was performed using a Thermo Scientific Nicolet TM iS10 model (USA) equipped with a diamond crystal. The spectra were measured within the range of 500–4000 cm^−1^ to analyze functional groups present on the surface of hydrochar and activated hydrochar.

Brunauer, Emmett and Teller (BET) (Autosorb1, Quantachrome, AsiQwin, USA) was used to determine the surface area, pore size and pore volume of each material by a multipoint technique using N_2_ adsorption at 78 K. The material was degassed at 180 °C for six hours.

### Batch adsorption study

Equilibrium and kinetic study parameters were determined by batch adsorption experiments. Activated hydrochar (0.03 g) and 30 mL solutions of cadmium concentrations ranging from 5, 10, 15, 20, 25 and 30 mg/L were added at neutral solution pH. The experiment was achieved in a thermal shaker at a given room temperature of 30 ± 2 °C for 4 h at 200 rpm. The mixtures were then left overnight to reach equilibrium. The material was filtered, and the filtrate was analyzed for cadmium concentration using flame atomic absorption spectrometry. The cadmium amount adsorbed on activated hydrochar was determined by Eq. (3).

Langmuir and Freundlich equilibrium isotherms were evaluated to best fit isotherm models with experimental data and explain the adsorption of cadmium. Kinetic study of the adsorption process represents the adsorbate uptake rate that controls the residual time of the complete adsorption process^14^. Kinetic adsorption models pseudofirst and pseudosecond order were used, according to the equations developed by Lagergren^33^) and Ho and McKay^34^.

### Cadmium (II) equilibrium adsorption isotherms

An equilibrium study was performed to examine the adsorption capacity and equilibrium relationship between the adsorbate and adsorbent. The experiment defines at constant temperature the ratio between the adsorbed amount and the remaining amount in solution at equilibrium.

Langmuir linear Eq. (4), as discussed above, was used to fit the experimental data for cadmium adsorption at equilibrium. The adsorbate forms monolayer coverage with homogeneous sites on the adsorbent surface.

Langmuir isotherm essential features are also expressed as dimensionless constants known as separation _factors (RLs_), also known as equilibrium parameters. It is expressed by Eq:

5 $$R_{L} = \frac{ 1}{{1 + bCo}}$$

Shape of the isotherm in terms of R_L_ either irreversible (R_L_ = 0), unfavorable (R_L_ > 1) or favorable (0 < R_L_ > 1).

The Freundlich isotherm model defines multilayer adsorption of the adsorbate on a heterogeneous system of the adsorbent. This model is expressed by Eq:

6 $$\text{log}\, q_{e} = {\text{log }}K_{f} { } + (1/n){\text{log }}Ce$$

where Ce shows the equilibrium concentration in mg/L and $q_{e}$ represents the cadmium amount adsorbed at equilibrium in mg/g. $K_f$ and n both represent Freundlich constants related to the adsorption capacity of the solid and the intensity of adsorption.

The Dubinin–Radushkevich (D–R) isotherm mainly describes the adsorption mechanism in terms of physical and chemical adsorption nature. D–R model The important factor is calculated by the following expressions:

7 $$ln \,qe=\mathrm{l}n\, qm-\beta \varepsilon^{2}$$

8 $$\varepsilon =RT\, \mathrm{l}n\, (1+ \frac{ 1}{Ce} )$$

9 $$E=\frac{ 1}{\sqrt{-2\beta }}$$

where β is the D–R constant and $\varepsilon$ is the Polanyi potential. R represent general gas constant (8.31 J/mol/K), T is the temperature in Kelvin, and E shows the mean adsorption energy in kJ/mol.

### Kinetic modeling

The adsorption process is based on kinetic studies, limits the residual time during adsorption and represents the uptake rate of the adsorbate. Equilibrium time is important to assess metal adsorption. Different solutions of 10 mg/L were added to 0.03 g adsorbent under optimized conditions and shaken vigorously for 5, 10, 15, 30, 60, 90, 120 and 150 min to determine the binding capacity and investigate the equilibrium time.

Kinetic sorption in terms of pseudofirst- and pseudosecond-order sorption is described by the following equations:

10 $$log \left( {qe - qt} \right) = \log qe - \frac{{K_{1} t}}{2.303}$$

11 $$\frac{t}{qt} = \frac{1}{{K_{2} q^{2} e}} + \frac{t}{qe}$$

qt and qe represent adsorption capacities (mg/g) at time (min). k_1_ and k_2_ are the rate constants for pseudofirst order and pseudosecond order. Parameter values obtained from their intercept and slope after plotting a linear graph of log (qe–qt) vs time for pseudofirst order and t/qt vs time for pseudosecond order.

### Desorption experiment

Loaded activated hydrochar was used to determine the desorption of cadmium. Deionized water and 0.01 M HNO_3_ were used as desorption agents. Dried cadmium-adsorbed activated hydrochar (0.5 g each) was added to a conical flask containing 20 mL of each of deionized water and 0.01 M HNO_3_. The experiment was achieved in a thermal shaker at room temperature (30 ± 2 °C) for 4 h at 200 rpm. The material was filtered to measure the cadmium concentration leached in the solution by flame atomic absorption spectrometry.

### Fixed-bed column adsorption study and modeling

Continuous fixed-bed column design experiments measure dynamic behavior to remove cadmium ions from aqueous solution. The experiment was carried out using a glass column with a height of 30 cm and an internal diameter of 1 cm. Glass wool (0.05 cm thick) was used above and below the adsorbent to provide mechanical support and to prevent any loss. The cadmium working solution was pumped by a peristaltic pump (Gilson MINIPULS ® 3) that maintained the desired continuous flow rate. Effluent was collected at different intervals of time up to 100 min, and the breakthrough point and saturation point were examined in an S-shaped breakthrough curve. The cadmium effluent concentration was analyzed by flame atomic absorption spectrometry. The time needed for a specific breakthrough concentration (usually 10% of the influent concentration) is called the breakthrough point (t_*b*_), and the saturation point is the time for the effluent concentration to influence 90% of the influent concentration where no more adsorption takes place. Breakthrough curve was obtained by C_*t*_/C_o_ against time^35^. For breakthrough curve analysis, the effects of bed height (1.3, 2.4 and 4.4 cm), flow rate (6, 9 and 12 mL/min) and cadmium concentration (10, 15 and 20 mg/L) were evaluated. Column performance was examined to calculate the breakthrough time with adsorption capacity. Breakthrough curve experimental studies were also compared with kinetic mathematical models Thomas, Yon-Nelson and Adam-Bohart. The dynamic experiment was performed at room temperature (30 ± 2 °C). The study was carried out at neutral pH because our focus was to remove cadmium ions from drinking water samples.

The total adsorbed quantity in milligrams was calculated with a specific flow rate with concentrations equivalent to the area underneath integrate plotting of adsorbed concentration C_ads_ (C_ads_ = C_0_–C_t_)^36^. Total adsorbed quantity was calculated by given Eq:

12 $$q_{total} = \frac{QA}{{1000}} = \frac{Q}{1000}\mathop \smallint \limits_{t = 0 }^{{t = t_{total} }} \left( {Co - Ct} \right) dt$$

where Q and A are the flow rate in mL/min and area of breakthrough curves, respectively. Co and Ct represent influent and effluent concentrations, and t_*total*_ is the total flow time used in minutes.

The total adsorbed amount of cadmium (m_*total*_) in mg was calculated by the following equation:

13 $$m_{total} = \frac{{Co . Q . t_{total} }}{1000}$$

cadmium total removal (%) is the ratio of adsorbed amount of cadmium to the total amount and was calculated by following Eq:

14 $$Total\,\, removal\,\, of \,\,cadmium\, \left( {II} \right) \,\,ions\, \left( \% \right) = \frac{{q_{total} }}{{m_{total} }} \times 100$$

The equilibrium metal uptake capacity (q_eq_) in mg/g is the adsorbed amount of cadmium per unit weight of sorbent calculated by Eq:

15 $$q_{eq} = \frac{{q_{total} }}{m}$$

where m shows the adsorbent mass in g that is packed in the column.

### Adam–Bohart model

Adam-Bohart explains breakthrough curve data at the breakpoint or 10% of the saturated point^37^. The rate of adsorption is proportionally related to the residual capacity of the sorbent with the concentration of adsorbing species^38^. Model equation is expressed as:

16 $$Where\;\;\;\;\;\;\;\;\;\;\;ln\left( {\frac{{C_{t} }}{{C_{o} }}} \right) = K_{AB} C_{o} t - K_{{{\text{AB}}}} { }{\text{.}}No\left( {\frac{Z}{{U_{o} }}} \right)$$

where, k_AB_ shows Adam–Bohart constant (L/mg min). N_0_ represents the maximum volumetric saturation concentration (mg/L), and Z is the bed depth of the column (cm). Linear flow velocity (Uo) in cm/min calculated from the flow rate over the fixed-bed cross-sectional area. Values of No and K_AB_ obtained by linear plotting of ln(Ct/Co) vs time in min.

### Thomas model

This model predicted column performance in terms of breakthrough curve experimental data^39^. It followed Langmuir kinetics adsorption and affected insignificant axial dispersion in the fixed-bed column, and the rate of driving force followed the second-order reversible kinetic reaction^40^. Thomas equation is expressed as

17 $$ln\left[ {\frac{{C_{o} }}{{C_{t} }} - 1} \right] = \frac{{k_{Th} q_{o} m}}{Q} - k_{Th} C_{o} t$$

q_o_ and k_Th_ represent the maximum solid phase equilibrium concentration (mg/g) and Thomas rate constant (mL/min mg), respectively. Parameter values are calculated by a linear plot of ln[(C_o_/C_t_) − 1) vs time in minutes.

### Yoon–Nelson model

Yoon-Nelson explained the adsorption process in a fixed-bed column. It assumes that a decrease in the probability rate during the adsorption process for adsorbate is proportional to the probability of adsorbate sorption and the probability of adsorbate breakthrough on the sorbent^41^. It is expressed in Eq:

18 $$ln\left( {\frac{{C_{t} }}{{C_{o} - C_{t} }}} \right) = k_{YN} . t - \tau . k_{YN}$$

k_YN_ is the Yoon–Nelson proportional constant (min^–1^), and $\tau$ is the time needed for 50% adsorbate breakthrough in minutes. The k_YN_ and $\tau$ values are obtained by plotting ln (Ct/(C_o_–C_t_) vs time in min.

## Data Availability

All data generated or analyzed during this study are included in this published article.
